# Supplementary material for: Altered microRNA Transcriptome in Cultured Human Liver Cells upon Infection with Ebola Virus
Source: Int J Mol Sci. 2021 Apr 6;22(7):3792. doi: 10.3390/ijms22073792 (PMC8038836; doi:10.3390/ijms22073792)
Supplement: Supplementary file 1 [file ijms-22-03792-s001.zip › Supplementary_File/C_ GO_Analysis_Results/16-30nt_go_Reston-96h-Huh7_vs_Control-96h-Huh7_down.mature_mirna_targets/CC_EnrichmentScoreDotPlot.pdf]

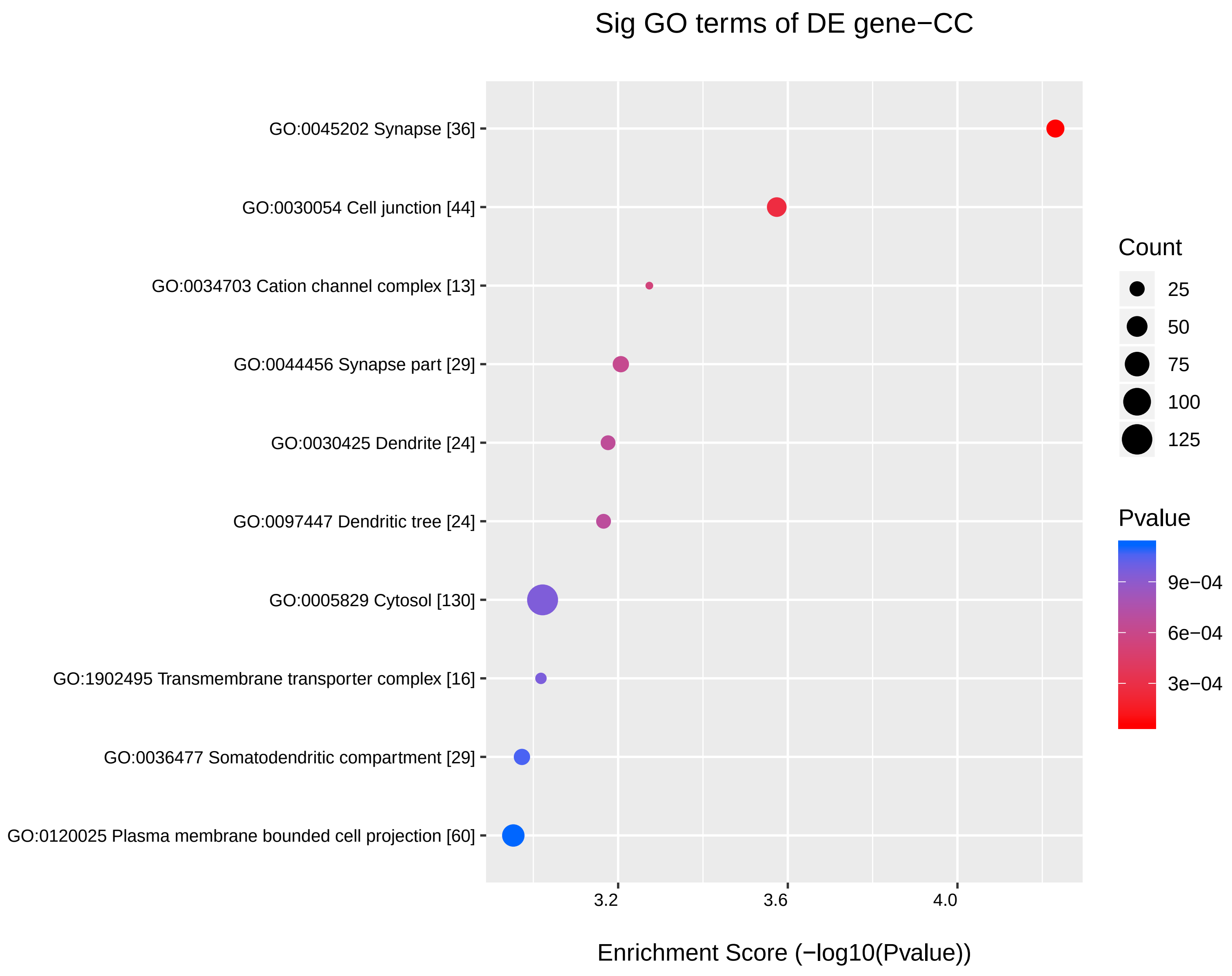
Sig GO terms of DE gene−CC
GO:0045202 Synapse [36]
GO:0030054 Cell junction [44]
GO:0034703 Cation channel complex [13]
GO:0044456 Synapse part [29]
GO:0030425 Dendrite [24]
GO:0097447 Dendritic tree [24]
GO:0005829 Cytosol [130]
GO:1902495 Transmembrane transporter complex [16]
GO:0036477 Somatodendritic compartment [29]
GO:0120025 Plasma membrane bounded cell projection [60]
3.2
3.6
4.0
Enrichment Score (−log10(Pvalue))
Count
25
50
75
100
125
Pvalue
9e−04
6e−04
3e−04
